# MicroRNA Expression and Intestinal Permeability in Children Living in a Slum Area of Bangladesh

**DOI:** 10.3389/fmolb.2021.765301

**Published:** 2021-12-08

**Authors:** Humaira Rashid, Towfida J. Siddiqua, Biplob Hossain, Abdullah Siddique, Mamun Kabir, Zannatun Noor, Masud Alam, Mamun Ahmed, Rashidul Haque

**Affiliations:** ^1^ Emerging Infections and Parasitology Laboratory, International Centre for Diarrheal Disease Research, Dhaka, Bangladesh; ^2^ Nutrition and Clinical Service Division (NCSD), International Centre for Diarrheal Disease Research, Dhaka, Bangladesh; ^3^ Department of Biochemistry and Molecular Biology, University of Dhaka, Dhaka, Bangladesh

**Keywords:** intestinal permeability, intestinal barrier dysfunction, qPCR, cytokines, fecal miRNA

## Abstract

**Introduction:** MicroRNAs (miRNAs) are small, non-coding RNAs that post-transcriptionally regulate gene expression. Changes in miRNA expression have been reported in a number of intestinal diseases, in both tissue samples and readily accessible specimens like stools. Pathogenic infections, diet, toxins, and other environmental factors are believed to influence miRNA expression. However, modulation of miRNAs in humans is yet to be thoroughly investigated. In this study, we examined the expression levels of two human miRNAs (miRNA-122 and miRNA-21) in stool samples of a group of Bangladeshi children who had an altered/increased intestinal permeability (IIP).

**Methods:** Stool samples were collected from children with IIP (L:M > 0.09) and normal intestinal permeability (NIP) (L:M ≤ 0.09). Quantitative PCR was performed to quantify the levels of miRNA-122 and miR-21 in stools. Commercial ELISA kits were used to measure gut inflammatory markers Calprotectin and REG1B. Serum samples were tested using Human Bio-Plex Pro Assays to quantify IL-1β, IL-2, IL-5, IL-10, IL-13, IFN-γ, and TNF-α. Total nucleic acid extracted from stool specimens were used to determine gut pathogens using TaqMan Array Card (TAC) system real-time polymerase chain reaction.

**Results:** The expression levels of miRNA-122 (fold change 11.6; *p* < 0.001, 95% CI: 6.14–11.01) and miR-21 (fold change 10; *p* < 0.001, 95% CI: 5.05–10.78) in stool were upregulated in children with IIP than in children with normal intestinal permeability (NIP). Significant correlations were observed between stool levels of miR-122 and miR-21 and the inflammatory cytokines IL-1β, IL-2, IFN-γ, and TNF-α (*p* < 0.05). Children with IIP were frequently infected with rotavirus, *Campylobacter jejuni*, *Bacteroides fragilis*, adenovirus, norovirus, astrovirus, and various *Escherichia coli* strains (ETEC_STh, ETEC_STp, EAEC_aaiC, EAEC_aatA) (*p* < 0.001). miR-122 significantly correlated with the fecal inflammatory biomarkers REG1B (*p* = 0.015) and Calprotectin (*p* = 0.030), however miR-21 did not show any correlation with these fecal biomarkers.

**Conclusion:** miR-122 and miR-21 may be associated with intestinal permeability. Assessment of these miRNAs may provide new tools for diagnosis of IIP in children from developing countries suffering diseases associated with intestinal barrier dysfunction.

## Introduction

The fundamental function of the intestinal tract is to absorb and digest nutrients. Additionally, apart from being the largest immune organ, the intestinal tract is also the largest reservoir of endotoxins and bacteria. The intestinal epithelium functions as a barrier that separates the body from the outside environment (Vancamelbeke and Vermeire, 2017). The most essential function of intestinal epithelial cells is maintenance of the intestinal epithelial barrier (Ulluwishewa et al., 2011). Disruption of the intestinal epithelial barrier is associated with inflammation and disturbance of the immune system. Earlier research indicated that toxins, pro-inflammatory cytokines, *in vitro* mild irritants and pathogens open the tight junctions and increase paracellular permeability (Fukui, 2016). Enteric pathogens also play a significant role in disruption of the epithelial barrier. Pathogens may stimulate immune and intestinal epithelial cells to secrete pro-inflammatory cytokines, including interleukin-1β (IL-1β), tumor necrosis factor-α (TNF-α), and interferon-γ (IFN-γ). These pro-inflammatory cytokines can induce intestinal barrier dysfunction and increase intestinal permeability (Bruewer et al., 2003). Intestinal epithelial barrier dysfunction has been documented to affect the children of low- and middle-income countries (LMIC), such as Bangladesh. Repeated chronic exposure to fecal pathogens and toxins can induce intestinal inflammation, which allows inflammatory molecules such as unwanted toxins, colonic bacteria and bacterial antigens to cross the barrier and intensify the immune response (Bischoff et al., 2014; Martinez-Medina and Garcia-Gil, 2014; Nishida et al., 2018). In Bangladesh, children who suffer persistent infections caused by astrovirus, adenovirus, rotavirus, norovirus, enterotoxigenic *Escherichia coli*, enteric protozoa (amebiasis, cryptosporidiosis, giardiasis), *Campylobacter jejuni* or *Bacteroides fragilis* are at risk of a sharp decline in growth and impaired nutritional status (Mondal et al., 2012; König et al., 2016; Prendergast and Kelly, 2016; George et al., 2018; Bray et al., 2019). These findings suggest that pathogen colonization may induce chronic inflammation and trigger changes in the intestinal microbiota that lead to disruption of the intestinal barrier (Buret et al., 2002; De Magistris et al., 2003; Mondal et al., 2012; Bischoff et al., 2014).

MicroRNAs (miRNAs) are short, highly conserved, non-coding 18–23 nucleotide RNA molecules that bind to the untranslated regions (UTRs) of mRNAs to control gene expression at the post-transcriptional level (Ha and Kim, 2014). The miRNAs function as post-transcriptional regulators by binding to their complementary sequences, which results in translational repression or degradation of specific mRNAs (O'Brien et al., 2018). As miRNAs regulate many important physiological processes in eukaryotic cells, their altered expression has been linked to a variety of diseases. This can lead to remarkable alterations to gene expression, and thus induce inflammatory conditions (Cichon et al., 2014). Intense exposure to enteric pathogens during infection induces inflammation, which allows pathogens to directly adhere to and invade the intestinal epithelial barrier, which in turn disrupt the tight junctional protein complexes composed of proteins such as occludin and claudins, leading to the onset and progression of intestinal diseases (Lee, 2015). MiRNAs have been shown to play crucial roles during infection with viruses such as the adenovirus, enterovirus, rotavirus, which are mainly responsible for gastroenteritis as well as changes in enterocyte and bacterial microflora (Maudet et al., 2014; Das et al., 2016; Huang et al., 2018; Mukhopadhyay et al., 2021).

In addition, a recent study reported that miRNAs are relevant to intestinal-associated disorders induced by infection and impact the regulation of inflammatory and immune responses (Sheedy and O’Neill, 2008; Bi et al., 2009). For instance, miRNAs, such as miR-21 and miR-122 target negative regulators of the immune response to promote inflammation. Expression of miR-122 and miR-21 have been reported to be dysregulated in patients with increased intestinal permeability (IIP) induced by various inflammatory stimuli (Zhang et al., 2015; Zhang et al., 2017). miR-21 is a ubiquitously expressed miRNA that is, traditionally considered as oncogenic (Li et al., 2012). However numerous miRNAs are commonly altered during pathogen infection and also induced by TNF-α and other cytokine molecules (Staedel and Darfeuille, 2013; Zhang et al., 2014; Drury et al., 2017). Persistent exposure to multiple enteropathogens induces a rapid change in miR-122 and miR-21 expression in enterocytes. Previous research indicates that children with enteric dysfunction caused by pathogen-induced inflammation have altered intestinal permeability, implying that inflammatory bowel syndrome (IBS) and other inflammatory-related gut pathologies may be exacerbated through such barrier dysfunction (Shulman et al., 2008).

The present study aimed to evaluate whether persistent exposure to multiple fecal pathogens leads to IIP and decreased barrier function that cause aberrant expression of miRNA-21 and miRNA-122. We also investigated whether aberrant levels of miRNA-122 and miRNA-21 can also influence the risk of intestinal inflammation. We assessed children living in communities where sanitation is often poor and hygiene practices are sub-optimal. IBS-like symptoms are widely accepted to persist in a small percentage of patients after a documented episode of intestinal viral or bacterial infection. Also, dysregulation of miRNAs has been implicated in several pediatric non-neoplastic diseases (Omran et al., 2013). Therefore, from our study we can determine that aberrant expression of microRNAs in children with an IIP can play a pathogenic role in diseases, including those primarily affecting the gut (Hollander et al., 1986; Distrutti et al., 2016).

## Materials and Methods

### Study Area and Human Subjects

This study was performed as a nested observational study (2016–2019) within a longitudinal birth cohort study in Mirpur slum areas of Dhaka city, Bangladesh (Kirkpatrick et al., 2015). A total of 442 children aged 2-years-old were selected for this study. Inclusion criteria for the current study were: mothers willing to sign informed consent form, no obvious congenital abnormalities or birth defects. The exclusion criteria were children aged below or above 2 years-of-age, children who do not have any major congenital anomalies and parents who are not willing to have child’s blood drawn.

### Sample and Data Collection

Urine samples were collected from the children (*n* = 442) over 5 h after ingestion of lactulose and mannitol solution. The selection of children for miRNA expression analysis was based on the availability of fecal samples from a previously conducted larger study. We assessed approximately 120 mg stool samples from 85 randomly selected children. From IIP group we selected 43 children and from NIP group we selected 42 children for miRNA-122 gene expression analysis and identical stool samples from 36 children (16 children with IIP and 20 children with NIP) for miRNA-21 gene expression analysis. However, remaining 49 children were not included for the miRNA-21 gene expression analysis because they did not have adequate stool volume to perform qPCR. The fecal biomarkers REG1B and Calprotectin were also assessed in the same stool samples used to determine miRNA-122 and miRNA-21. From the same children we also analyzed 3-ml blood samples to quantify inflammatory cytokines and correlate with miRNA-122 and miRNA-21**.** Diarrheal stools were assessed to detect enteropathogens. The urine, blood and stool samples were transported from the field clinic to the Parasitology Lab, Icddr,b in a cold box and samples were stored at −70°C prior to analysis.

### Anthropometry

Anthropometric measurements were taken at the time of enrolment using a calibrated digital baby scale (UC-321; A & D Co., Tokyo, Japan) and standardized supine length measurement equipment (TALC, St Albans, Herts, UK) to determine height-for-age (HAZ) z-score and weight-for-age (WAZ) z-scores, measures of stunting and underweight, respectively (World Health Organization, 1995). A HAZ less than −2 SD is defined as stunting and a WAZ less than −2 SD is defined as underweight.

### Measures of Intestinal Permeability

The L/M ratio is a test of intestinal permeability. Individuals with intestinal barrier dysfunction exhibit increased absorption of lactulose due to increased mucosal permeability and decreased absorption of mannitol due to a decrease in the intestinal surface area (Zhang et al., 2000). The lactulose and mannitol was given to the children at a dose of 2 ml/kg of body weight. The solution in water included 250 mg/ml of lactulose and 50 mg/ml of mannitol. Urine was collected upto 2 h after lactulose and mannitol ingestion. The assay was conducted using high-performance anion exchange chromatography (HPAEC-PAD). Children with a lactulose to mannitol ratio (L:M > 0.09) were considered to have increased IIP and children with a (L:M ≤ 0.09), NIP (Musa et al., 2019).

### MicroRNA Extraction in Stool Specimens

Given that miRNAs have previously been detected in other body fluids (Cortez et al., 2011), we compared the presence of miRNAs in the fecal samples of children with IIP and NIP.

Total RNA was extracted from the stool samples using miRNAeasy Mini Kits (Qiagen). Stool (120 mg) was mixed with RNAse free water and homogenized using a vortex mixer in Qiazol lysis reagent for 30 s. RNA was precipitated by adding chloroform into a 2 ml RNAse-free tube, vortexed for 15 s, incubated at room temperature for 2–3 min, centrifuged at 12,000 g for 15 min at 4°C and total stool RNA was eluted in RNase free water. Finally, the RNA concentration was measured using a Nanodrop 2000 (Thermo Fisher Scientific, Wilmington, DE, United States).

### Reverse Transcription and Real-Time PCR

The Revert Aid First Strand cDNA Synthesis Kit (Thermo Scientific, United States) was used to perform reverse transcription (RT) according to the manufacturer’s instructions. A RT universal stemloop primer and total treated RNA was used to perform the RT reaction (Raymond et al., 2005). Each 20 μL RT reaction mixture contained 0.1‒5 μg/1 μL treated total RNA, 1 μL of stem-loop RT primer (5 μM), 1 μL of 10 mM dNTP blend, 4 μL of reaction buffer, 1 μL of Ribolock RNase inhibitor (20 U/μL), and 1 μL of Revert Aid MMuLV Reverse Transcriptase (200 U/μL). The mixtures were incubated at 65°C for 5 min, then at 42°C for 60 min, and stopped by heating to 70°C for 5 min. The cDNA was stored at −20°C until further use.

SYBR Green [EXPRESS SYBR^®^ GreenER™ qPCR SuperMix Universal (Invitrogen)] was used to quantify the miRNAs in the stool nucleic acid samples. Reverse-transcribed RNA was quantified using a Bio-Rad CFX96 real-time PCR detection system. A 12 µL PCR reaction volume included 5 µL of SYBR green super mix, 1 µL of RT product and 0.4 µL of primer (forward and reverse, 1 µM each). The reactions were incubated at 50°C for 2 min, 95°C for 2 min, followed by 40 cycles of 95°C for 15 s, 60°C for 60 s. The expression levels of miRNA were measured using the quantification cycle values (Cq values). U6 RNA was used as an endogenous control/housekeeping gene for data normalization and the assay was quantified by the 2^−△△Cq^ method (Livak and Schmittgen, 2001; Bustin et al., 2009; Jacobsen et al., 2011). The 2^−ΔΔCT^ method [ΔΔCT = ΔCT (a miRNA of interest)-ΔCT (U6 RNA as a normalizer accounting for sample-to-sample variation)] was used to analyze the relative expression of miRNA-122 and miRNA-21.

### TaqMan Array Card for the Detection of Enteric Pathogens

Total nucleic acid extracted from stool samples was used to detect multiple enteropathogens using The TaqMan Array Card system real-time polymerase chain reaction format by Applied Biosystems ViiA7 Real-Time PCR system (Life Technologies, Foster City, CA, United States), which can rapidly detect and quantify 27 enteropathogens, including helminths and viruses. TAC enables the identification of a wide range of enteropathogens in a fast and precise manner with a quantitative detection.

### Stool Enzyme-Linked Immunosorbent Assay

Fecal Calprotectin a gut inflammatory biomarker for intestinal mucosal inflammation and gut damage (Harper et al., 2018) was measured following the manufacturer’s instructions by using commercially available ELISA kits (Calprotectin ELISA, BÜHLMANN Laboratories AG, Basel, Switzerland) (Jensen et al., 2011). Fecal Calprotectin was quantified using a standard curve generated using the kit standards and expressed as µg/g stool.

Another inflammatory biomarker fecal REG1B which is a potential indicator of intestinal injury and repair (Peterson et al., 2013) was also measured using ELISA technique (TECHLAB, Inc., Blacksburg, VA, United States) to quantify REG1B concentration (µg/g) in stool samples to evaluate epithelial health.

### Cytokine Measurements

The serum samples were tested for IL-1β, IL-2, IL-5, IL-10, IL-13, IFN-γ, and TNF-α using Human Bio-Plex Pro Assays (Bio-Rad, Hercules, CA) (Jiang et al., 2014) on a Bio-Plex 200 platform (Bio-Rad, Hercules, CA, United States). Bio-Plex Manager software version 6.0 was used for data analysis.

### Statistical Analysis

Statistical analyses were conducted using SPSS (version 17.0) or GraphPad prism 8.0 software (San Diego, CA, United States). Results were expressed as mean ± standard deviation (SD). Differences were considered significant if *p* ≤ 0.05. Data were compared using Mann–Whitney *U* tests and *t*-test was applied to test the association of miRNAs and pathogenic infections with intestinal permeability. Bivariate correlations between variables, including miRNA levels, cytokines and fecal inflammatory biomarkers, were examined using Pearson’s correlation coefficient (r). The sensitivity and specificity of miRNAs for identifying children with IIP (L:M > 0.09) were calculated by receiver operating characteristic (ROC) curve analysis.

## Results

### Nutritional Status of Children

A total of 442 children were available for this study; 47.2% of the children were female (Table 1) and 37.5% (166/442) had elevated L:M ratio (> 0·09) suggestive of increased intestinal permeability (IIP). Of these 442 children, 30.3% of the children were less than −2 WAZ and 32.3% were less than −2 HAZ.

**TABLE 1:** Clinical and demographic features of children in Mirpur slum area, Dhaka, Bangladesh. Moderate underweight −3 ≤WAZ < −2, severely underweight WAZ < −3.

Characteristics (442 children)	Number	Percentage
Age in years	2	—
Female	209	47.2
Moderate underweight	111	25.1
Severely underweight	23	5.2
Moderate stunted	102	23
Severely stunted	41	9.2

Moderate stunted −3 ≤HAZ < −2, severely stunted HAZ < −3.

42% (69/166) of children with IIP were less than −2 WAZ while only 23.5% (65/276) with normal intestinal permeability (NIP) children were less than −2 WAZ which is statistically significant (*p* = 0.0001). Similarly, 40% (66/166) of children with IIP were less than −2 HAZ whereas, 27.8% (77/276) children with NIP were less than −2 HAZ which is also statistically significant (*p* = 0.012). We also checked for the nutritional status of the selected 85 children for miRNA-122 expression analysis; 27% children were less than −2WAZ and 28.2% children were less than −2HAZ.

### miR-122 and miR-21 Expression Levels in Stool

**FIGURE 1:**
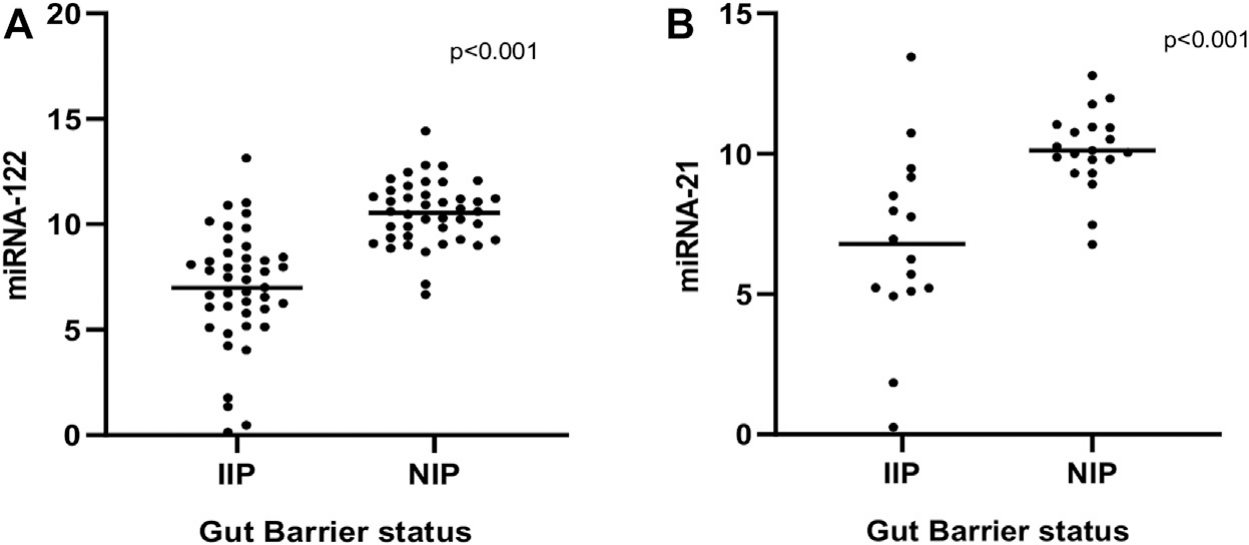
Comparison of expression levels of miRNA-122 **(A)** and miRNA-21 **(B)** between normal and increased intestinal permeability respectively. Statistical analysis was carried out by the Mann-Whitney *U* test.

miR-122 and miR-21 expression analyzed from the fecal RNA where the total RNA concentration was from 661 to 2,298 ng/μL. Data derived from RT-qPCR are presented as normalized ∆CT values. A significant differential expression was determined in stool samples of children with IIP compared to NIP children for miR-122 (*p* < 0.001). Similarly, fecal samples of children with IIP showed significant differential expression of miR-21 compared to children with NIP (*p* < 0.001). The mean ± standard deviation (SD) normalized ct values for miR-122 and miR-21 were significantly different in children with IIP compared to children with NIP (miR-122: 6.99 ± 2.75 vs. 10.53 ± 1.51; miR-21: 6.78 ± 3.24 vs. 10.12 ± 1.40). miR-122 and miR-21 expression levels of stool were upregulated 11.6; (*p* < 0.001, 95% CI: 6.146–11.01) and 10; (*p* < 0.001, 95% CI:5.055–10.78) times respectively in children with an increased intestinal permeability when compared to children with a normal intestinal permeability. These results are graphically presented in Figure 1.

### Receiver Operating Characteristic Curve Analysis for miRNA-122 and miRNA-21

**FIGURE 2:**
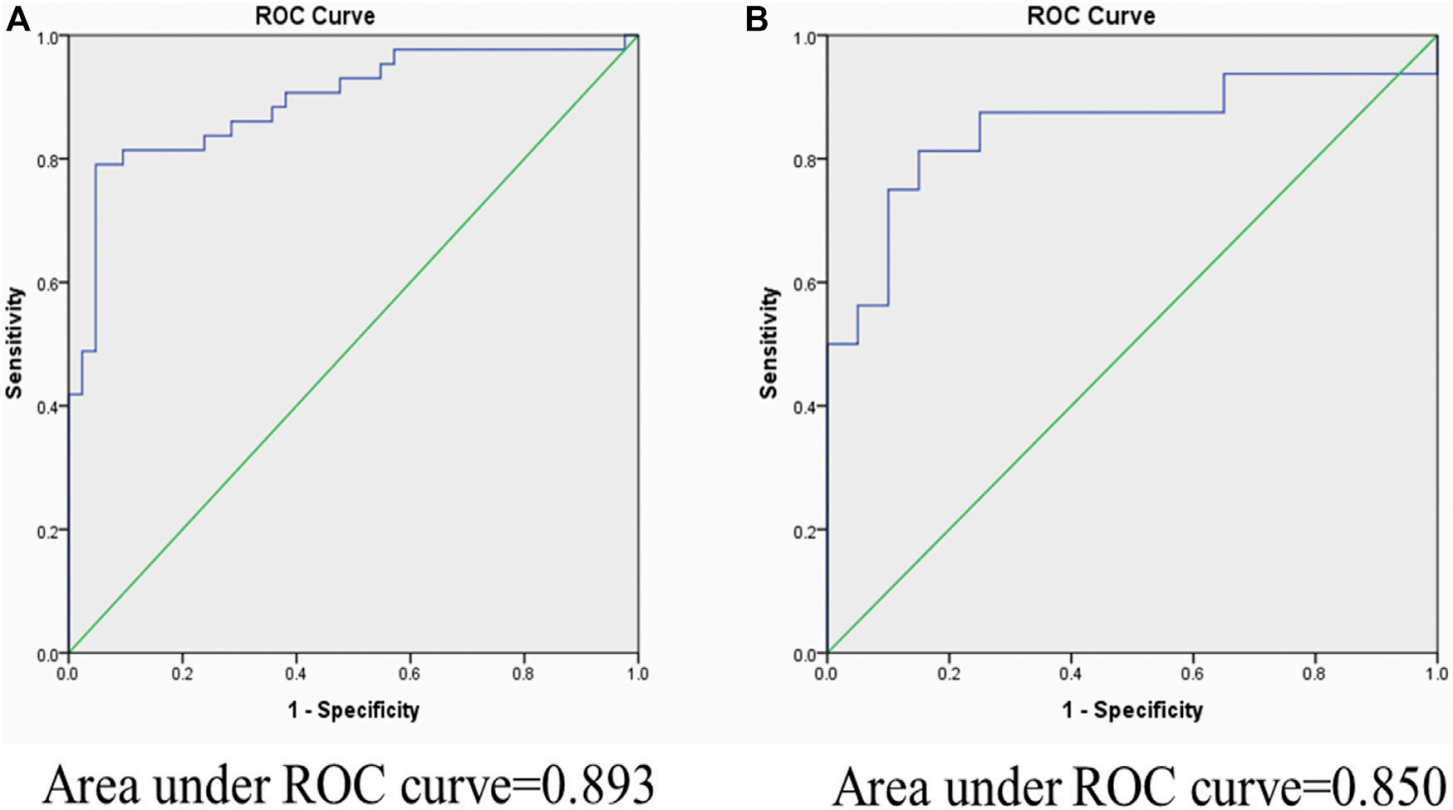
Area under curve (AUC) of receiver operating characteristic (ROC) analysis for miR-122 and miR-21 relative expression in fecal samples for discriminating increased and normal intestinal permeability. ROC curve analysis revealed that fecal miR-122 and miR-21 relative expression has significant specificity and sensitivity to distinguish between children with IIP and NIP. **(A)**: miR-122 yield an area under the curve (AUC) value of 0.893 (*p*-value = 0.0001). **(B)**: miR-21 yield an area under the curve (AUC) value of 0.850 (*p*-value = 0.0001).

Biomarkers are important for early disease identification, as early diagnosis can help to avoid and improve prognosis. Stool-based miRNA detection has high sensitivity and specificity (Yau et al., 2019). To investigate the possibility that these miRNAs may serve as new and potential biomarkers for increased intestinal permeability, receiver operating characteristic (ROC) curve analysis was carried out to evaluate the diagnostic potential of stool miRNA-122 and miRNA-21 levels. As shown in Figure 2, the AUC values for miR-122 and miR-21 were 0.893 [95% confidence interval (CI) 0.822–0.964, *p* = 0.0001] and 0.850 [95% confidence interval (CI) 0.706–0.94, *p* = 0.0001], respectively. This result demonstrated that miR-122 and miR-21 have potentials for detection of IIP. Thus, the stool miRNA-122 and miRNA-21 levels could be considered as promising biomarkers for the diagnosis of IIP in children.

We also compared the stool miRNA expression levels with the L:M ratios of the children to identify cut-off values of miR-122 and miR-21 that indicate IIP. A L:M ratio >0.09 is regarded as increased intestinal permeability and a L:M ratio ≤0.09 is regarded as normal intestinal permeability. Using a cut-off of 9.8 for miR-122 (normalized ct value) 84% (36/43) of children with IIP were below this cut-off. Thus, at this cut-off value miRNA-122 had a sensitivity of 84% and specificity of 71.4% to detect IIP. Using the same cut-off value for miR-21, 88% (14/16) children with IIP were below this cut-off, with a sensitivity of 88% and specificity of 75% (Tables 2A,B).

**TABLE 2:** **(A)** Normalized miR-122 ct value (DCT) *Lactulose: Mannitol ratio (L:M) Status Crosstabulation. L:M status 1 indicates children with (L:M > 0.09) considered to have increased IIP and L:M status 0 indicates children with (L:M ≤ 0.09), considered to have NIP. DCT 1 indicates normalized miR-122 ct value ≤9.8 and DCT 2 indicates normalized miR-122 ct value >9.8. **(B)** Normalized miR-21 ct value (DCT)*Lactulose: Mannitol ratio (L:M) status crosstabulation. L:M status 1 indicates children with (L:M > 0.09) were considered to have increased IIP and 0 indicates children with (L:M ≤ 0.09), were considered to have NIP. DCT 1 indicates normalized miR-21 ct value ≤9.8 and DCT 2 indicates normalized miR-21 ct value >9.8.

Count				
		L:M status (Lactulose: Mannitol status)		Total
		0	1	
DCT (normalized miR-122 ct value)	1	12	36	48
	2	30	07	37
Total		42	43	85

Count				
		L:M status (Lactulose: Mannitol status)		Total
		0	1	
DCT (normalized miR-21 ct value)	1	05	14	19
	2	15	02	17
Total		20	16	36

### Association of Enteric Pathogens With Intestinal Permeability

We compared the presence of enteric pathogens in children with IIP and children with NIP. Rotavirus, *Campylobacter jejuni, Bacteroides fragilis*, adenovirus, norovirus, astrovirus, and *Escherichia coli* strains (*Enterotoxigenic Escherichia coli*-STh, Enterotoxigenic *Escherichia coli*-STp, Enteroaggregative *Escherichia coli*-_aaiC, Enteroaggregative *Escherichia coli*-aatA) were significantly more frequent in children with IIP compared to children with NIP (*p*-value < 0.001) (Supplementary Figure).

### Correlation of Inflammatory Cytokines With miRNAs

**FIGURE 3:**
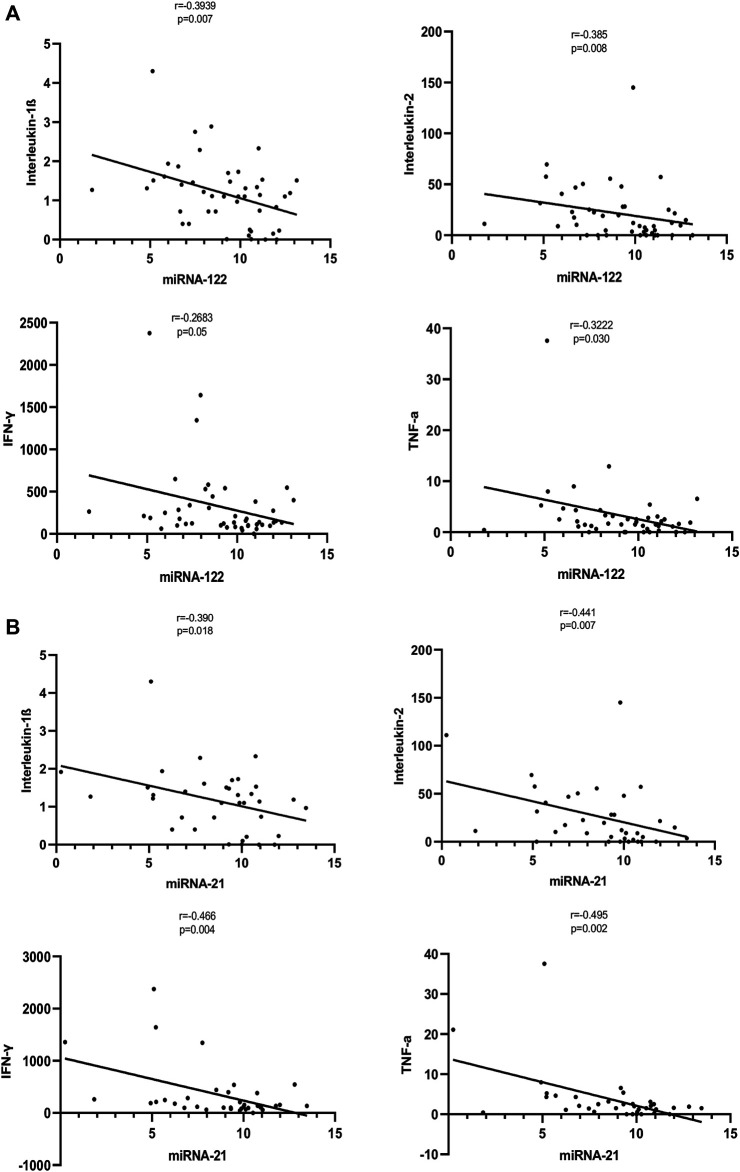
**(A)**: Correlation of expression levels of miRNA-122 with inflammatory cytokines. The *X*-axis indicates the miR-122 relative expression, while the *Y*-axis denotes cytokines. We performed the Pearson’s correlation test for statistical evaluation. **(B)**: Correlation of expression levels of miRNA-21 with inflammatory cytokines. The *X*-axis indicates the miR-21 relative expression, while the *Y*-axis denotes cytokines. We performed the Pearson’s correlation test for statistical evaluation.

We assessed the correlations between the inflammatory cytokines (IL-1β, IL-2, IL-5, IL-10, IL-13, IFN-γ, and TNF-α), with the levels of miRNAs (miR-122 and miRNA-21). The cytokines IL-1β, IL-2, IFN-γ, and TNF-α levels were correlated significantly with the levels of the miRNAs. The expression level of miRNA-122 correlated with the levels of IL-1β (*r* = −0.3939, *p* = 0.007), IL-2 (*r* = −0.385, *p* = 0.008), IFN-γ (*r* = −0.2683, *p* = 0.05), and TNF-α (*r* = −0.3222, *p* = 0.030) The expression level of miRNA-21 correlated with the levels of IL-1β (*r* = −0.390, *p* = 0.018), IL-2 (*r* = −0.441, *p* = 0.007), IFN-γ (*r* = −0.466, *p* = 0.004) and TNF-α (*r* = −0.495, *p* = 0.002) (Figures 3A,B). No significant correlations were observed between miRNA levels (miR-122 and miRNA-21) with inflammatory cytokines IL-5, IL-10, or IL-13.

### Correlations Between miRNA-122 and miRNA-21 and Fecal Inflammatory Biomarkers

**FIGURE 4:**
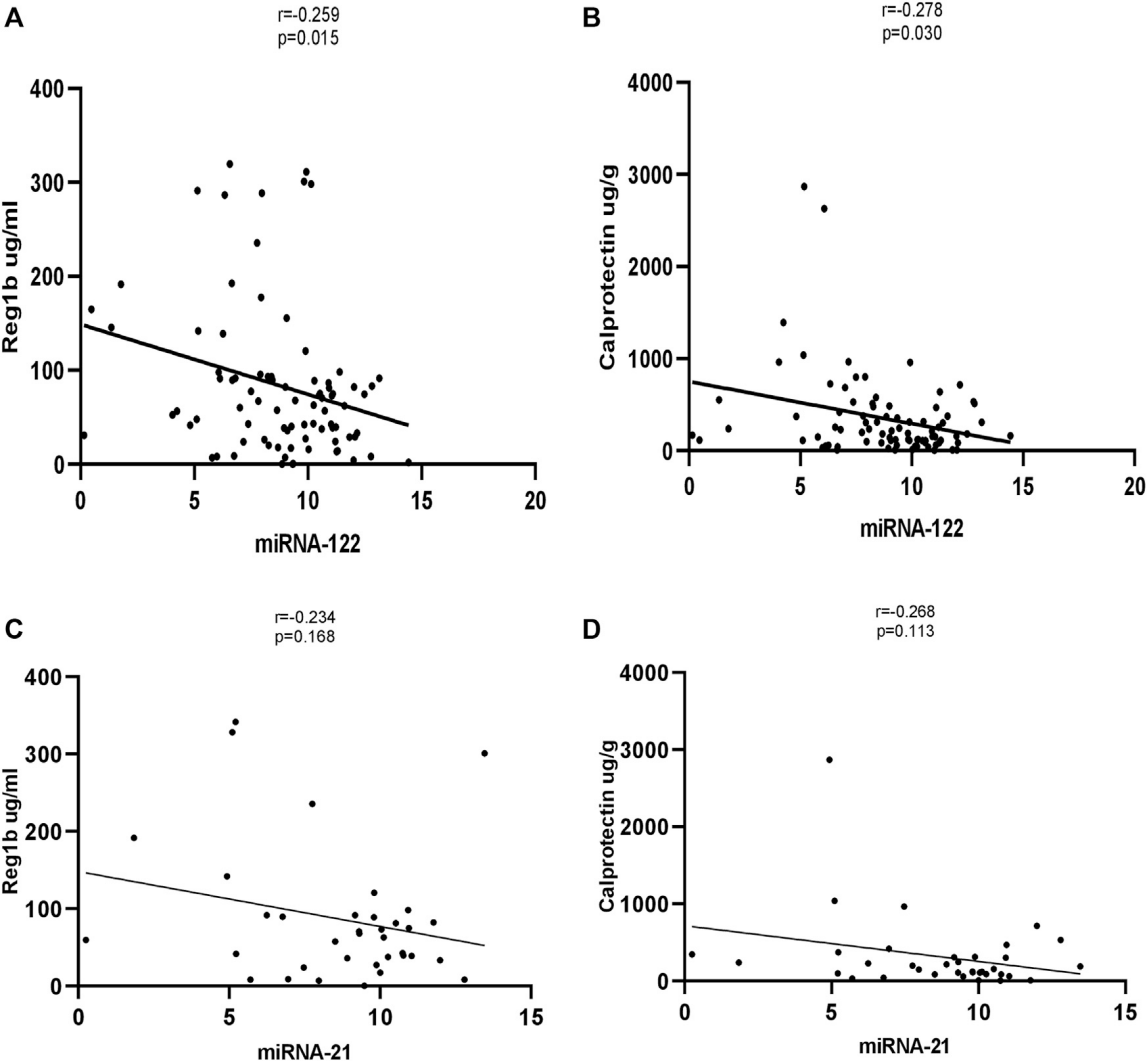
Correlation of expression levels of miRNA-122 and miRNA-21 values with fecal biomarkers (with Reg1b and Calprotectin). The *X*-axis indicates the relative expression of miR-122, while the *Y*-axis denotes fecal biomarkers. We performed Pearson’s correlation test for statistical evaluation which showed a significant association between miRNA-122 **(A, B)** and fecal biomarkers, but no significant association was seen between miRNA-21 and fecal biomarkers **(C, D)**.

Significant correlations were observed between miR-122 and REG1B. The stools of children with a high concentration of REG1B contained higher expression levels of miRNA-122 (*r* = −0.259, *p* < 0.015) compared to children with low concentration of REG1B. Calprotectin, the most common inflammatory biomarker observed during disease progression, correlated significantly with miRNA-122 (*r* = −0.278, *p* < 0.030). However, both REG1B and Calprotectin fecal biomarkers were not correlated significantly with miR-21 (Figure 4).

## Discussion

Exposure to various enteric pathogens causes extensive mucosal disruption (Kosek et al., 2017) and intestinal inflammation leading to IIP and microbial translocation. We assessed intestinal permeability among 442 children using the lactulose/mannitol assay of urine samples; 166 children had a L:M ratio >0.09, indicative of altered/increased intestinal permeability. Previous studies associated IIP with the presence of enteric pathogens, suggesting a mechanism of intestinal and systemic inflammation (Amour et al., 2016; Rogawski et al., 2017). In our study, we demonstrated that the expression levels of miR-122 and miR-21 were significantly upregulated in children with IIP compared to children with normal intestinal permeability.

Compared to children with normal intestinal permeability, miRNA-122 (fold change 11.6; *p* < 0.001) and miR-21 (fold change 10; *p* < 0.001) were significantly upregulated in children with IIP. Similarly, previous research identified that unique miRNA expression profile signatures in intestinal-related diseases and persistent exposure to multiple fecal pathogens lead to IIP and decreased barrier function, which may cause aberrant expression of miRNA-122 and miRNA-21 (Ye et al., 2011; Zhang et al., 2015; Johnston et al., 2018; Al-Sadi et al., 2020; Rashid et al., 2020). Zhang et al. (2015) observed significant overexpression of miRNA-21 was associated with increased tight junction permeability *in vitro.* Another study showed the levels of several miRNAs, including miR-21, miR-122, miR-155, miR-146a, miR-429, and miR-874, were altered in small intestinal epithelial cells isolated from patients in a burns unit with IIP (Morris, 2018; Zhang et al., 2018) found that miRNA-21 is upregulated during intestinal barrier dysfunction (Ye et al., 2011; Liu et al., 2019). Reported that miRNA-122 and miRNA-21 may also play an important role in the maintenance of intestinal barrier function, thus dysregulation of miRNA-122 and miRNA-21 can lead to altered intestinal barrier function.

The levels of specific miRNAs have also been reported to be modulated in the feces of patients affected by enteric pathogens (Liu et al., 2016; Liu and Weiner, 2016; Sarshar et al., 2020), suggesting these miRNAs may be involved in the pathogenesis of intestinal barrier dysfunction and could potentially represent diagnostic or prognostic tools for altered intestinal permeability/intestinal barrier dysfunction (Zhou et al., 2010; Zhang et al., 2015; Zhang et al., 2017). In our study, a number of pathogens were detected in children with altered intestinal permeability. Adenovirus, rotavirus, different strains of *Escherichia coli* (ETEC_STh, ETEC_STp, EAEC_aaiC, EAEC_aatA), *Campylobacter. jejuni*, *Bacteroides. fragilis*, norovirus, and astrovirus were frequently detected in the stool samples of children with IIP. The presence of pathogens can affect miRNA expression, which in turn may disrupt the intestinal barrier. This suggestion is in line with a growing body of evidence that indicates enteric pathogens can induce intestinal inflammation by modifying the intestinal microbiota, weakening the intestinal barrier, and priming the intestine for chronic inflammatory responses (Amour et al., 2016; Kosek et al., 2017; Fahim et al., 2018; Fahim et al., 2020). Moreover, the expression of miRNAs can change dramatically during the course of infection with pathogens (Zhao et al., 2015; Drury et al., 2017). Infections with viruses such as rotavirus are one of the most common causes of diarrhoea in children <5 years, and no effective drug is available to treat these viruses. One study showed that oral administration of a mimic miRNA-7 agomir inhibited the replication of rotavirus in mice, indicating miR-7 may suppress the replication of rotavirus (Zhou et al., 2020). In another study, compared to controls children infected with adenovirus showed altered microRNA expression profiles (Huang et al., 2018). Similarly, as notable findings of this study, infections caused by enteric pathogens were associated with aberrant levels of miRNA-122 and miRNA-21, which could have a profound impact on the development and growth of children. These fecal miRNAs are related to the dysbiosis and inflammation of the host. Dysbiosis of the normal intestinal microbiota can lead to a number of diseases including inflammatory bowel diseases (Tlaskalová-Hogenová et al., 2011). In addition, miRNAs have been found to play a vital role in modulating pathogenic infections, and are primarily responsible for alterations to the bacterial microflora (Schönauen et al., 2018). Thus, the potential of miRNAs as tools for genetic research, diagnosis and treatment of pathogenic infections caused by bacteria, viruses, parasites, and fungi in human merits further investigation.

Expression of REG1B and Calprotectin are elevated in tissue samples from patients with benign diseases, such as acute amoebic colitis (Peterson et al., 2011), Crohn’s disease and ulcerative colitis (Lawrance et al., 2001; Bjarnason, 2017). In one study, the levels of fecal miRNAs correlated with fecal Calprotectin levels (Schönauen et al., 2018). Interestingly, in this study, the fecal biomarker REG1B—a putative measure of intestinal injury and repair—correlated significantly with the levels of miRNA-122 in stool samples. Also, Calprotectin—the most commonly assessed inflammatory biomarker of disease progression—significantly correlated with miRNA-122. However, both REG1B and Calprotectin fecal biomarkers was not statistically significant with miR-21, most likely due to low subject numbers.

This study examined a population in which undernutrition, as well as pathogen exposure, are prevalent. One study showed that Escherichia *coli* is characterized by destruction of epithelial cells and are associated with an upregulation of cytokines such as TNF-α and IFN-γ (Shea-Donohue et al., 2010). Our results indicate that although the prevalence of IBD is lower in Bangladesh than among children in western countries, the high infection burden induces increased expression of inflammatory cytokines, which leads to aberrant miR-21 and miR-122 expression among children with IIP. Furthermore, numerous published studies suggest a significant link between miRNAs and cytokine activities, since miRNA expression is regulated in response to cytokine stimulation. Furthermore, our analysis suggests that the *C*_t_ values of miR-122 and miR-21 in stools negatively correlate with the serum levels of the inflammatory cytokines TNF-α, INF-γ, IL-1β, and IL-2 (r < 0) where low *C*_t_ values indicates higher expression of miRNA-122 and miRNA-21. These findings are in line with (Chen et al., 2014; Yan et al., 2020) who found that miR-21 and miR-122 can promote the production of inflammatory cytokines that are closely related to the pathogenesis of IBD, such as TNF-α, IFN-γ, and IL-1β. In general, miRNA-122 and miRNA-21 target negative regulators of the immune response to promote inflammation. Several studies found that miRNA-21 and miRNA-122 exerted inflammatory roles in intestinal barrier dysfunction that leads to impaired barrier function (Ye et al., 2011; Zhang et al., 2015; Zhang et al., 2017). Showed that TNF-α can abruptly upregulate miRNA-122 and miRNA-21 in enterocytes and intestinal tissues, and consequently lead to barrier disruption and IIP.

To our knowledge, no previous studies have examined the expression levels of miRNA-122 and miRNA-21 in the fecal samples of Bangladeshi children or explored the association of these miRNAs with IIP and the presence of enteric pathogens. The findings of this study provide critical evidence that dysregulation of miRNAs during inflammation could be potentially involved in the pathogenesis of intestinal-related diseases. Furthermore, the correlations between these miRNAs and other inflammatory biomarkers offers new possibilities to use miRNAs as disease biomarkers.

However, this study has limitations. Firstly, correlation between fecal inflammatory biomarkers REG1B and Calprotectin and miRNA-21 did not show a statistical significance. However, the correlation was seen to be significant with miRNA-122. We also did not see any correlation between fecal pathogens and miRNA-122 and miRNA-21. This could be due to small sample size that limits statistical power to detect differences. Secondly, this study was conducted in children residing in the slum areas of Mirpur, Bangladesh. To validate these findings, large clinical studies in different geographical regions are needed. Our findings suggest fecal miRNA-21 and miRNA-122 may have potential as clinically useful, non-invasive prognostic and diagnostic biomarkers for pediatric patients, though this suggestion requires confirmation in large validation groups. Additional studies with larger sample sizes are required to reliably validate our findings. Also, our data point towards a molecular mechanism by which dysregulation of miRNAs due to altered intestinal permeability can lead to impaired growth/malnutrition, and correction of the levels of miRNAs using therapeutics could potentially help to manage environmental enteric dysfunction.

In conclusion, we show that Bangladeshi children with IIP have substantially altered levels of miRNAs in their stools. Two fecal miRNAs were closely linked to disease activity and easily accessible surrogate biomarkers, such as REG1B, fecal Calprotectin, and the serum levels of inflammatory cytokines. Furthermore, this study demonstrates that the levels of miRNAs in feces are very stable and can be reproducibly detected, even after long term storage. Overall, in conjunction with previous findings the levels of miRNAs in feces correlate with disease activity, our results indicate miRNAs merit further research as potential biomarkers of gut barrier diseases.

## Data Availability

The original contributions presented in the study are included in the article/Supplementary Material, further inquiries can be directed to the corresponding author.
