# Supplementary figures and images for: MicroRNA Expression and Intestinal Permeability in Children Living in a Slum Area of Bangladesh
Source: Front Mol Biosci. 2021 Dec 8;8:765301. doi: 10.3389/fmolb.2021.765301 (PMC8692878; doi:10.3389/fmolb.2021.765301)

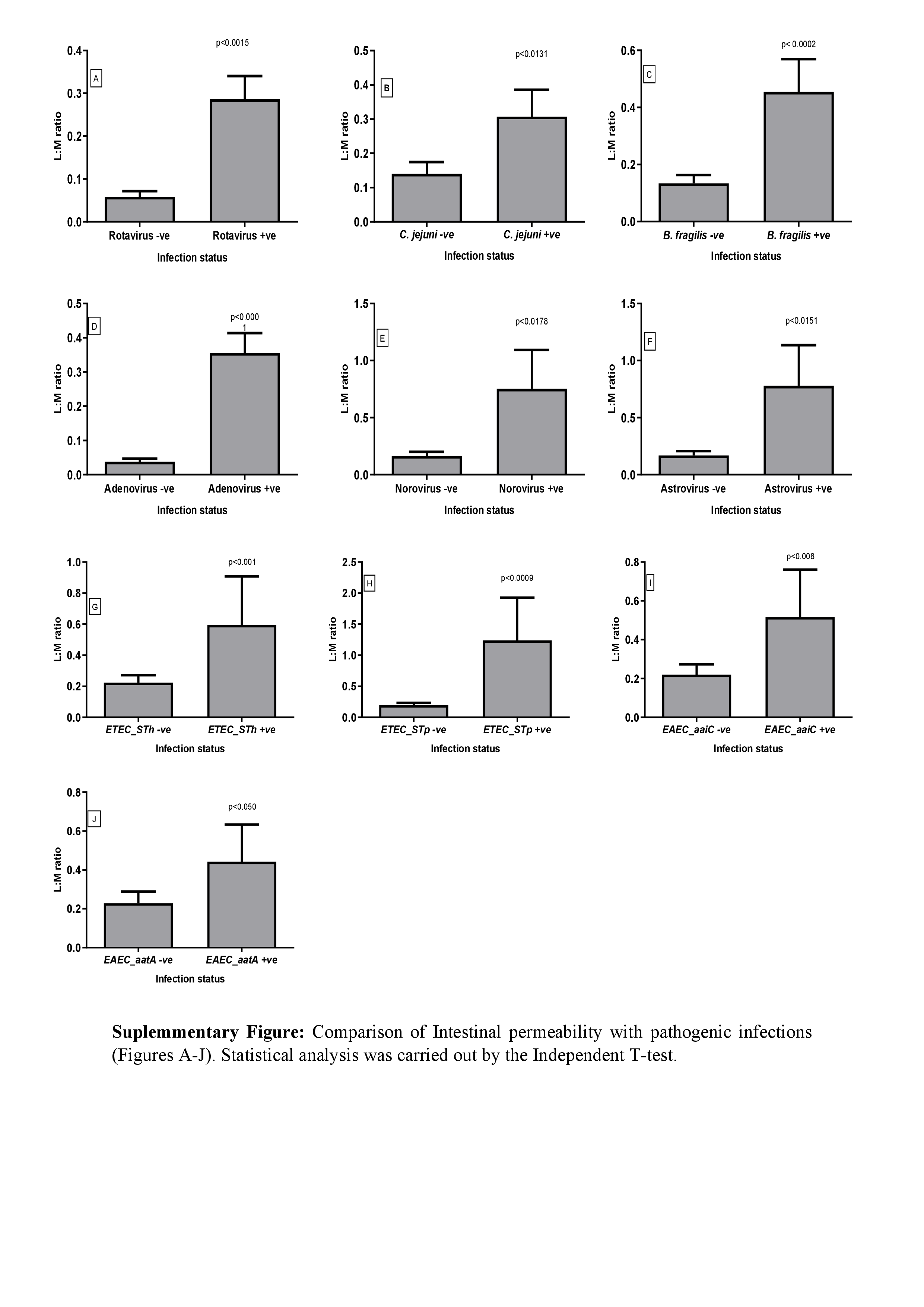

Supplement: Supplementary file 1 [file Image1.TIFF]
