# Supplementary material for: MicroRNA Expression and Intestinal Permeability in Children Living in a Slum Area of Bangladesh
Source: Front Mol Biosci. 2021 Dec 8;8:765301. doi: 10.3389/fmolb.2021.765301 (PMC8692878; doi:10.3389/fmolb.2021.765301)
Supplement: Supplementary file 2 [file Table1.DOCX]

**Supplementary Table : Primers used for quantitative RT-PCR.**

| RNA | 5' to 3' |
| --- | --- |
| hsa-miR-122 | Forward 5' - GGGAACGCCATTATCACAC - 3' |
|  | Universal Reverse 5' - GTGCAGGGTCCGAGGT - 3' |
| U6 | Forward 5′- CTCGCTTCGGCAGCACA-′3 |
|  | Reverse 5′-AACGCTTCACGAATTTGCGT-′3 |
| hsa-miR-21 | Forward 5′-GTTTGGTAGCTTATCAGACTGA-′3 |
|  | Universal Reverse 5′-GTGCAGGGTCCGAGGT-′3 |
|  |  |
|  |  |
